# Effectiveness of Nutritional Supplements for Attenuating the Side Effects of SARS-CoV-2 Vaccines

**DOI:** 10.3390/nu15081807

**Published:** 2023-04-07

**Authors:** Paola Gualtieri, Domenico Trombetta, Antonella Smeriglio, Giulia Frank, Angela Alibrandi, Giulia Leggeri, Marco Marchetti, Ilaria Zingale, Silvia Fanelli, Arianna Stocchi, Laura Di Renzo

**Affiliations:** 1 Section of Clinical Nutrition and Nutrigenomic, Department of Biomedicine and Prevention, University of Tor Vergata, Via Montpellier 1, 00133 Rome, Italy; 2 Department of Chemical, Biological, Pharmaceutical and Environmental Sciences, University of Messina, Viale Ferdinando Stagno d’Alcontres 31, 98166 Messina, Italy; 3 Ph.D. School of Applied Medical-Surgical Sciences, University of Tor Vergata, Via Montpellier 1, 00133 Rome, Italy; 4 Department of Economy, University of Messina, Via dei Verdi 75, 98122 Messina, Italy; 5 School of Specialization in Food Science, University of Tor Vergata, Via Montpellier 1, 00133 Rome, Italy

**Keywords:** immunonutrient, nutritional supplement, vaccine side effects, SARS-CoV-2

## Abstract

Supplementation is known to enhance the immune response and reduce infection. Therefore, the association between immune nutrients and vaccine side effects needs to be investigated. Our aim was to analyze the relationship between vaccination side effects and supplement intake among the Italian population. The study included a questionnaire asking for personal data, anthropometric information, COVID-19 infection and immunity response, and COVID-19 vaccination and supplementation. The survey was conducted from 8 February to 15 June 2022. In the study, 776 respondents were included, aged between 18 and 86 (71.3% females). We observed a statistically significant correlation between supplement consumption and side effects at the end of the vaccination cycle (*p* = 0.000), which was also confirmed by logistic regression (*p* = 0.02). Significant associations were observed between supplement intake and side effects of diarrhea and nausea at the end of the vaccination cycle (*p* = 0.001; *p* = 0.04, respectively). Significant associations were observed between side effects and omega-3 and mineral supplementation at the start of the vaccination cycle (*p* = 0.02; *p* = 0.001, respectively), and between side effects and vitamin supplementation at the end of the vaccination cycle (*p* = 0.005). In conclusion, our study shows a positive impact of supplementation on vaccination response, increasing host immune defenses, and reducing side effects.

## 1. Introduction

The occurrence of the SARS-CoV-2 virus and the establishment of a global pandemic led to the adoption of preventive economic and health measures to address the devastating impact of the virus. COVID-19 infections primarily affect the upper and lower respiratory tracts and induce a strong innate and adaptive immune response, which, although initially serving a protective role, later leads to a rapid exacerbation of the inflammatory response. Consequently, there was a rapid worsening of the patients’ clinical conditions. Indeed, when the inflammatory response is overexcited, a dysregulation of the pro-inflammatory cytokine release occurs, altering the physiological homeostasis [1].

This imbalance between pro-inflammatory cytokines such as interleukin (IL)-1, IL-6, and tumor necrosis factor-alpha (TNF-α), and the anti-inflammatory response leads to an acute systemic inflammatory syndrome, namely cytokine release syndrome (CRS), characterized by fever and multiple organ dysfunction [2].

Since the genetic sequence of the SARS-CoV-2 virus was published on January 11, 2020, scientists, industries, and other organizations around the world have collaborated to develop, as soon as possible, safe and effective vaccines against COVID-19, with the aim of producing an immune response able to neutralize the virus and prevent cell infections [3]. Some vaccines have been made using the same technology as vaccines currently in use; others have been made using new approaches [4]. Studies on vaccines against COVID-19 started in spring 2020, and in less than one year, the European Medicines Agency (EMA) has authorized the conditional marketing of a first messenger RNA (mRNA) vaccine, namely Comirnaty (BNT162b2), from the BioNTech/Pfizer company, and immediately after, a second one produced by Moderna, namely Spikevax. Like Comirnaty, the Spikevax (mRNA-1273) vaccine is based on mRNA technology. The latter encodes the spike (S) protein of the SARS-CoV-2 virus. The vaccine, therefore, does not introduce the virus into the cells but only the genetic information that the cell needs to build copies of the S protein [3,5]. Following the positive opinion of the EMA, in January 2021, the third COVID-19 vaccine, Vaxzevria (ChAdOx1-S), developed by the University of Oxford and AstraZeneca, was authorized for marketing by the European Commission. Compared to the first two mRNA vaccines, Vaxzevria uses a different approach to induce the body’s immune response to the S protein. It is a viral vector vaccine that uses a modified version of the chimpanzee adenovirus, which is no longer able to replicate, as a vector to provide instructions for synthesizing the S protein of SARS-CoV-2. Once produced, the protein can stimulate a specific immune response (both antibody and cellular) [5]. Finally, on 11 March, 2021, the EMA recommended the granting of a conditional marketing authorization for the vaccine developed by the pharmaceutical company Janssen (Johnson & Johnson). It is a viral vector vaccine composed of a recombinant vector based on replication-incompetent human adenovirus type 26 suitably modified to contain the gene encoding the complete S protein sequence of the SARS-CoV-2 virus in a stabilized conformation (Ad26.COV2.S). 

A lower risk of developing adverse effects with mRNA vaccines compared to viral vector vaccines had already emerged from pre-marketing studies, which was also found post-marketing [5,6]. Considering this, it became important immediately to identify, quantify, and evaluate all the risks related to the administration of individual vaccines, carrying out the appropriate risk-benefit analyses [7]. Several studies have detected and analyzed post-vaccination symptoms, correlating them with the different types of vaccines and their mechanisms of action. The most frequent local adverse effects were pain, erythema, swelling, and lymphatic adenopathy, whereas the systemic ones were fever, headache, fatigue, myalgia, arthralgia, nausea, vomiting, diarrhea, and chills [8]. The least frequent but most serious adverse effects were instead lymphedema, myocarditis, Bell’s palsy, respiratory distress, anaphylaxis, and thrombosis with thrombocytopenia syndrome [9].

It is well known that the onset of side effects is often associated with a compromised immune response due to an unhealthy lifestyle, an unbalanced diet, and comorbidities [10]. On the contrary, an optimal nutritional status guarantees the modulation of inflammatory and oxidative processes related to the immune system, playing a fundamental role in counteracting bacterial and viral infections as well as being proven for SARS-CoV-2 infection [11]. Indeed, it has been demonstrated that an increase in the risk of COVID-19 infection, severe disease, and death were directly associated with diet-associated inflammation [12].

Recently, however, it has been found that some vitamins and microelements not only play a preventive role against SARS-CoV-2 infection but also support vaccine immunogenicity and efficacy [11]. Several studies are available from this point of view, so much so that recently, the key role that vitamins, such as vitamin A, B6, B12, C, D, E, K, and folate; microelements, such as zinc, iron, selenium, and copper; and nutrients, such as omega-3 (*ω*-3) polyunsaturated fatty acids, have in supporting innate and adaptive immune responses, have been extensively reviewed [13,14,15,16,17,18]. Preclinical and clinical studies support specific roles for dietary supplements containing them, especially regarding the maintenance of physical barriers in the skin, gastrointestinal, and respiratory tracts, as well as highlighting their key role in supporting cellular functionality in innate and adaptive responses [18]. Moreover, probiotics such as *Bifidobacteria* and *Lactobacilli* have a positive effect in modulating the dysbiosis of the gastrointestinal and pulmonary systems associated with the SARS-CoV-2 infection. Indeed, it has been demonstrated that the intestinal microbiota, through the bidirectional intestine-lung axis, influences the health status of the lungs [11]. In association with them, the integration of prebiotics is useful for modulating inflammation and promoting the growth of the intestinal microbiota, allowing an adequate immune response [19].

Although supplementation has been shown to enhance the immune response and reduce SARS-CoV-2 infection, to date, the association between dietary supplements and vaccine side effects has not been investigated.

Considering this, the aim of this study was to evaluate the relationship, both at the start and at the end of the vaccination cycle, between vaccination side effects and their duration as well as dietary supplement intake. For this purpose, the different types of vaccine as well as the participants who took at least one dose of vaccine or who had completed the mandatory vaccination cycle have been analyzed. The associations between dietary supplements, pathological status, lifestyle, level of education, territorial origin, and SARS-CoV-2 infection were investigated. Finally, results were stratified for subject age and gender difference to highlight any possible correlation.

## 2. Materials and Methods

The project “Eating habits and lifestyle changes during COVID-19 lockdown: an Italian survey″ (EHLC-COVID-19) [20] started in April 2020 by the Section of Clinical Nutrition and Nutrigenomic, Department of Biomedicine and Prevention of the University of Rome Tor Vergata. 

A web survey was conducted to obtain data on supplementation and COVID-19 vaccination side effects for EHLC-COVID-19 from 8 February to 15 June 2022, among the Italian population. The end date of the survey was established based on the achievement of a minimum number of respondents, calculated to achieve a statistical power higher than 80%.

It was administered on an online platform through institutional and private social networks (Facebook, Twitter, and Instagram) and institutional mailing lists, following the snowball model.

The inclusion criteria were as follows: age over 18 years and access to the Internet. Exclusion criteria were as follows: age under 18, drug use, psychotic disorders, neoplastic diseases under treatment, pregnancy, breastfeeding, and HIV/AIDS.

The questionnaire was specifically developed using Google Forms, accessible through any device with an Internet connection. The questionnaire included 48 questions divided into four different sections: (1) personal data (10 questions: sex at birth; age; region of residence; county of residence; current employment; degree; lifestyle; practiced sport; smoked; and suffered from some pathology); (2) anthropometrics information (2 questions: reported weight and height); (3) COVID-19 infection and immunity response (3 questions: contracted COVID-19; symptoms during the COVID-19; and differences in health status before and after COVID-19 infection); and (4) COVID-19 vaccination and supplementation (23 questions: vaccinations received; how many doses; type of vaccinations received; vaccination effects at the first, second, and third dose; duration of side effects; and separate, non-combined intake of supplements, represented by *ω*-3, vitamins (vitamin C, D, E, and B), minerals (zinc, selenium, and copper), and biotics (prebiotics and probiotics).

The SARS-CoV-2 vaccination cycle was considered complete after the mandatory doses, according to Italian law.

The full version of the questionnaire is available as Appendix A.

The retrospective observational population-based study was conducted in accordance with national and international regulations and the Declaration of Helsinki (2013). All participants were fully informed about the study requirements and were required to accept the data sharing and privacy policy according to the EU, GDPR No. 2016/679, before participating in the study. Participants completed the questionnaire while connected to the Google platform. Each participant’s personal information was anonymized to maintain and protect confidentiality. The anonymous nature of the web survey does not allow sensitive personal data to be traced. Therefore, this web survey does not require the approval of the Ethics Committee [20]. Once completed, each questionnaire was transmitted to the Google platform, and the final database was downloaded as a Microsoft Excel spreadsheet. 

### Statistical Analysis

Data are represented as numbers and percentages in parentheses (%) for categorical variables or median and interquartile range in square brackets [IQR] for continuous variables. The Shapiro–Wilk test was performed to evaluate the variable distribution. All the variables had a skewed distribution. The Spearman correlation coefficient was calculated to evaluate the correlation between continuous variables. The Chi-square test of independence was employed to assess the association between categorical variables, dividing the data from the answers to specific questions into the categories ‘YES’ and ‘NO’. Finally, binary and multinomial logistic regression analyses were conducted to investigate the association between categorical variables (dependent) and continuous or categorical variables (independent). Results were significant for a *p*-value < 0.05. Statistical analysis was performed using SPSS ver. 21.0 (IBM, Chicago, IL, USA).

## 3. Results

On 15 June 2022, the web survey was concluded, and the collected data were analyzed. Respondents aged between 18 and 86 were included in this study. We enrolled 776 subjects who completed the questionnaire to achieve a statistical power higher than 80%. In fact, assuming an incidence of nonserious adverse events in the population equal to 81.3% [21] and an incidence in our sample equal to 77% (considering an alpha significance level of 5%), the minimum number of subjects to be enrolled is equal to 676. 

Female respondents represent 71.3% of the population. For the vaccination cycle of three doses, 626 subjects completed it. Additionally, 139 received two doses, and 11 received only one dose. Regarding vaccination types, the following were administered at the start of the vaccination cycle: (1) Comirnaty to 522 subjects; (2) Spikevax to 71 subjects; (3) Vaxzevria to 154 subjects; and (4) Jcovden to 28 subjects. The following were administered at the end of the vaccination cycle: Comirnaty to 433 subjects and Spikevax to 224 subjects.

General characteristics and anthropometrics of the population are reported in Table 1.

**Figure 1:**
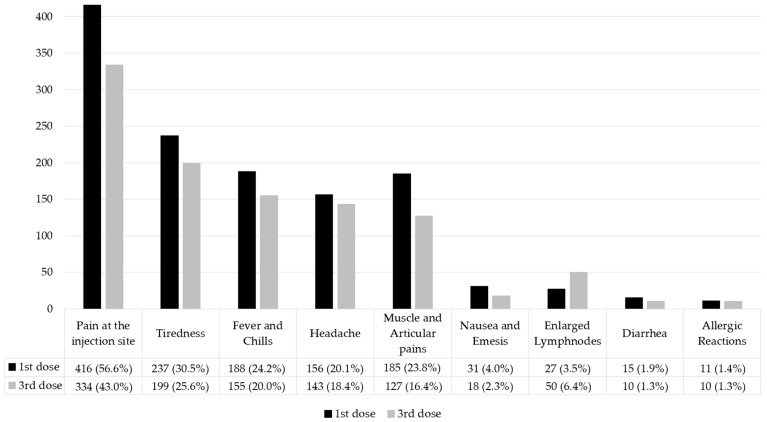
Vaccination side effects. Distribution of vaccination side effects at the start (1st dose) and end (3rd dose) of the vaccination cycle. Values are expressed as numbers and percentages (n (%)) for categorical variables.

The vaccination side effects are reported in Figure 1.

The main intakes of supplements during the vaccination cycle are reported in Table 2.

Differences by age were investigated. Considering the variables age, vaccine side effects, and supplementation, no significant correlation was found. A statistically significant correlation was observed between age and comorbidities (*p* = 0.000).

A significant correlation was observed between COVID-19 symptoms and the presence of comorbidities (*p* = 0.03). The correlation results are reported in Table 3.

Although we could not perform an analysis of the actual immune response to the vaccine, we did take into account if SARS-CoV-2 had been contracted after the vaccine doses. At the start of the vaccine cycle, no significant correlation was observed between sexes in the effect of supplementation on immune responses (SARS-CoV-2 contractors or not) (*p* = 0.78). 

At the start of the vaccine cycle, no significant correlation was observed between vaccination side effects and supplement intake (*p* = 0.98). No significant correlation was also observed between vaccination side effects and the presence of comorbidities (*p* = 0.38). The correlation results are reported in Table 4.

During the vaccine cycle, significant correlations were observed for sex differences in the effect of supplementation on immune responses (SARS-CoV-2 contractors or not). Particularly, significant correlations between the male sex and prebiotics (*p* = 0.01), probiotics (*p* = 0.01), and *ω*-3 (*p* = 0.048) supplementation were observed.

At the end of the vaccination cycle, a significant correlation was observed between vaccination side effects and supplement intake (*p* = 0.000). No significant correlation was observed between the side effects and the presence of comorbidities (*p* = 0.82). The correlation results between vaccination side effects, supplement intake, and comorbidities at the end of the vaccination cycle are reported in Table 5.

At the end of the vaccine cycle, significant correlations with sex differences in the effect of supplementation on immune responses (SARS-CoV-2 contractors or not) were observed. Particularly, significant correlations were observed for male sex and prebiotics (*p* = 0.047), *ω*3 (*p* = 0.01), and L-glutamine (*p* = 0.045) supplementation, and for female sex and vitamin D supplementation (*p* = 0.04). 

A logistic regression showed that higher supplement intake was associated with decreased vaccination side effects at the end of the vaccination cycle (*p* = 0.02; OR = 1.74; 95%CI 1.11–2.73) but not at the start (*p* = 0.85; OR = 0.96; 95%CI 0.65–1.42). 

A significant association was observed between supplement intake and vaccination side effects at the third dose of Comirnaty (X^2^ (1, N = 430) = 7.16, *p* = 0.008). Patients taking supplements (24.7%) are more likely to have no effects than those who did not take them (40.7%). The effect size measure was evaluated by Cramer’s V index and was equal to 0.13.

Chi-square tests were performed to analyze possible associations between individual side effects and supplement intake. Results are reported in Table 6. 

A significant association was observed between supplement intake and diarrhea at the start of the vaccination cycle (*p* = 0.007). Patients with supplement intake (19.5%) are more likely to have no diarrhea side effects than those who did not take them (80.5%) at the start of the vaccination cycle.

A significant association between male sex and fevers and chills (side effects) during the vaccination cycle was observed (*p* = 0.049). Males are more likely to have the side effects of fevers and chills than females during the vaccination cycle. No other significant associations between gender and side effects were observed at the start (*p* = 0.80), during (*p* = 0.69), or at the end (*p* = 0.25) of the vaccination cycle.

Significant associations were observed between supplement intakes and diarrhea and nausea at the end of the vaccination cycle (*p* = 0.001 and *p* = 0.04, respectively). Patients taking supplements (19.9%) are more likely to have no side effects of diarrhea and nausea than those who did not take them (80.1%) at the end of the vaccination cycle.

No other significant associations were observed between supplement intakes and individual side effects at the start, during, or at the end of the vaccination cycle.

After examining the distribution of the data regarding supplements, it was decided to aggregate the categories into ‘*ω*-3’, ‘Vitamins’ (vitamin C, D, E, and B), ‘Minerals’ (zinc, selenium, and copper), and ‘Biotics’ (prebiotics and probiotics) to facilitate the interpretation of the statistical analysis. Chi-square tests were performed to analyze possible associations between side effects and individual supplement intakes. Chi-square test results are reported in Table 7.

Significant associations were observed between side effects and *ω*-3 and mineral supplementation at the start of the vaccination cycle (*p* = 0.001 and *p* = 0.02, respectively). Patients with *ω*-3 (2.6%) and mineral (2.8%) supplementation are more likely to have no side effects than those who did not take it at the start of the vaccination cycle.

A significant association between side effects and vitamin supplementation at the end of the vaccination cycle was observed (*p* = 0.005). Patients taking vitamin supplementation (18.1%) are more likely to have no side effects than those who did not take it (81.9%) at the end of the vaccination cycle.

No other significant associations were observed between side effects and individual supplement intake at the start and end of the vaccination cycle.

## 4. Discussion

The main aim of this study was to evaluate the relationship between vaccination side effects and their duration and supplement intake, both at the start and end of the vaccination cycle and for individual vaccines, in participants who have had at least one dose of a vaccine or who have completed the entire vaccination cycle. Particularly, we observed a statistically significant correlation between supplement consumption and side effects at the end of the vaccination cycle (*p* = 0.000), which was also confirmed by logistic regression (*p* = 0.02, OR = 1.74; 95%CI 1.11–2.73). Significant associations were observed between supplement intake and side effects of diarrhea and nausea at the end of the vaccination cycle (*p* = 0.001; *p* = 0.04, respectively). Significant correlations were observed in male sex and prebiotics (*p* = 0.01), probiotics (*p* = 0.01), and *ω*-3 (*p* = 0.048) during the vaccination cycle. Significant correlations were observed between female sex and vitamin D supplementation (*p* = 0.04) and between male sex and prebiotics (*p* = 0.047), *ω*-3 (*p* = 0.01), and L-glutamine (*p* = 0.045) supplementation at the end of the vaccination cycle. For the first time, significant associations between side effects and *ω*-3 and mineral supplementation at the start of the vaccination cycle (*p* = 0.02; *p* = 0.001, respectively) were noted. Furthermore, a significant association was observed between side effects and vitamin supplementation at the end of the vaccination cycle (*p* = 0.005).

COVID-19 infections can develop in different forms; affected patients may be asymptomatic or symptomatic with mild or more severe symptoms. In the most critical cases, where hospitalization might be required, pathological damage can be found through infiltration and hyperplasia at the level of the alveolar tissue; this can lead to different deadly diseases such as heart failure, septic shock, and ARDS [22]. 

Furthermore, in subjects infected with SARS-CoV-2, the presence of multiple comorbidities characterized by a low-grade systemic inflammatory state (diabetes, cardiovascular diseases, obesity, neurodegenerative diseases, and cancer) can increase the response of the immune system, increasing the risk of adverse effects and mortality [23].

In agreement with D’Errico et al. [23], from our analysis, we observed a statistically significant correlation between the presence of comorbidities and the worsening of COVID-19 symptoms (*p* = 0.03). We also observed a statistically significant correlation between age and comorbidities (*p* = 0.000). Although Wagner et al. [24] observed how age can influence humoral and cellular immune responses after vaccination, we did not find significant correlations considering the variables age, vaccine side effects, and supplementation.

Vaccines have been a key point in stopping the global pandemic, despite numerous interfering variables, such as equitable global access to vaccines and the will of individuals [25].

At the end of the web survey, 776 respondents (18–86 years) were included in the study, and a prevalence of female respondents (71.3%) was found.

All participants were vaccinated (76.4% with mRNA vaccines), although the type of vaccine differed between the population and during the vaccination cycle. As the first dose, 67.3% received Comirnaty, 9.1% received Spikevax, 19.8% received Vaxzevria, and 3.6% received Jcovden. When Spikevax was withdrawn from commerce and Jcovden was globally used only for one dose, the vaccination cycle was completed for 65.9% of participants with Comirnaty and 34.1% of participants with Spikevax. Approximately 80.7% of participants completed the vaccination cycle, 17.9% received two doses, and 1.4% received only one dose.

Several side effects of individual SARS-CoV-2 vaccines have been reported. For example, Klugar et al. observed that injection site pain was the side effect most associated with the mRNA vaccine, while headache and fatigue were associated primarily with viral vaccines [26]. 

In our study, at the first dose, the participants who reported some side effects were 70.7% of the ones that received Comirnaty, 77.5% of the ones that received Spikevax, and 69.5% and 67.9% of the ones that received Vaxzevria and Jcovden, respectively. In particular, injection site pain was the most common side effect related to all vaccine types (32.5% for Comirnaty, 30.7% for Spikevax, 33.3% for Vaxzevria, and 44.7% for Jcovden).

At the end of the vaccination cycle, 61.9% and 89.7% of the participants who received Comirnaty and Spikevax, respectively, reported some side effects. Specifically, injection site pain was the most common side effect related to both vaccine types (34.5% for Comirnaty and 28.6% for Spikevax).

In the study by Almufty et al., conducted among the Iraqi population, statistically significant correlations were found between post-vaccination side effects and comorbidities, female gender, having contracted a previous infection with COVID-19, and young age [27]. 

Nassara et al. also observed that compromised health conditions (for example, the presence of autoimmune diseases) and regular intake of drugs (such as non-steroidal anti-inflammatory drugs) are factors that most influence possible post-vaccine side effects [28]. 

We did not observe a statistically significant correlation between the presence of comorbidities and vaccination side effects (*p* = 0.38). However, we cannot draw a conclusion regarding the correlation between the presence of comorbidities and vaccination side effects due to the low frequency of subjects with comorbidities (3% of the sample), which may need to be more statistically representative. 

In general, the individual’s response to vaccination is related to their immune, nutritional, and genetic status, even if 2–10% of the population does not respond adequately to vaccination [29].

In our study, we investigated micronutrient supplementation and its role in vaccination side effects. Particularly, we observed a statistically significant correlation between supplement consumption and side effects at the end of the vaccination cycle, specifically following the administration of Comirnaty (*p* = 0.008). A similar result was obtained with a logistic regression showing that increased supplement intake is associated with decreased side effects at the end of the vaccination cycle but not at the start (*p* = 0.85; OR = 0.96; 95%CI 0.65–1.42). Specifically, we observed significant associations between supplement intake and diarrhea and nausea side effects at the end of the vaccination cycle. These results are in agreement with Saresella et al. [30], who observed that the immune response increases with the administration of vaccine doses.

Furthermore, we observed significant correlations on the basis of sex differences in the effect of supplementation on immune responses during and at the end of the vaccination cycle but not at the start. Particularly, we noted a significant association between male sex and fevers and chills during the vaccination cycle (*p* = 0.049). These results are in agreement with Klein et al. [31], who noted how sex can influence innate and adaptive immune responses, leading to sex-specific outcomes even for infectious diseases and vaccines.

Micronutrient deficiency causes a reduction in antibody concentration, compromising natural killer (NK) cell cytotoxicity and cellular immunity and attenuating the response to vaccination. Therefore, micronutrients are indispensable not only for COVID-19 prevention and treatment [32], but also for the immune response that follows vaccination [33].

The importance of antioxidant-based supplementation lies in the different oxide reduction (redox) mechanisms that regulate both viral entry and cytosolic replication of coronaviruses. SARS-CoV-2 spike proteins preferentially utilize angiotensin-converting enzyme 2 (ACE2) as a receptor in airway epithelial cells [34], which plays a key role in the induction of oxidative stress [35]. The complex crosstalk between ACE2 and redox pathways is further emphasized by possible bidirectional redox regulation of ACE2 levels. High ACE2 activity can reduce oxidative stress, but conversely, high redox stress can regulate ACE2 [36,37,38,39].

In the present study, the role of supplement intake, represented by *ω*-3, vitamins (vitamin C, D, E, and B), minerals (zinc, selenium, and copper), and biotics (prebiotics and probiotics), was investigated to test the hypothesis that the intake of nutrients with antioxidant action could reduce the side effects of COVID-19 vaccination.

According to the literature, vitamin D, vitamin C, vitamin E, and selenium are powerful antioxidants, capable of counteracting reactive oxygen and nitrogen species, protecting cell membranes [19], and creating barriers in the respiratory tract [33]. Sharif et al. [40] observed a significant association between vitamin C, vitamin D, and zinc supplementation and reduced infection and severity of COVID-19 risk, as well as the duration of supplementation and medication being significantly associated with reduced hospitalization.

Regarding vitamin C supplementation in patients with COVID-19, several studies have been conducted, often leading to inconclusive and contrasting results [41,42,43]. Additionally, it has been observed that high-dose vitamin C could lead to a reduction in mortality and an improvement in oxygen support status. Furthermore, vitamin D supplementation in COVID-19 patients can lead to a reduction in pro-inflammatory cytokines, limiting the mortality associated with ARDS. Patients with deficiency were also observed to have an increased risk of pneumonia [44].

In our study, vitamins supplementation was taken by 26.8% of the participants at the start of the vaccination cycle, but it was reduced by 1.8% at the end. Furthermore, concerning the significant association between side effects and vitamin supplementation at the end of the vaccination cycle, it was observed that patients taking vitamin supplementation (18.1%) are more likely to have no side effects than those who did not take it (81.9%) at the end of the vaccination cycle. These results are in agreement with Newton et al. [45], who observed that vitamin A supplementation in children who had received the pentavalent vaccine leads to a reduction in fever and illness as side effects. 

It was observed that zinc supplementation leads to beneficial effects in counteracting the SARS-CoV-2 infection, improving immune function, increasing IL-2, IL-6, and IFN-γ, and decreasing IL-10. Furthermore, according to Heller et al. [46], the zinc biomarker could represent a good predictor of survival in COVID-19, highlighting that its supplementation is fundamental for convalescence in patients with deficiency.

Selenium supplementation in COVID-19 patients with deficiency is known to improve the course of infection [33], and, according to Hackler et al. [47], the selenoprotein P biomarker could be a fundamental survival parameter in COVID-19, showing that its supplementation is relevant for the convalescence of deficient patients. 

However, to date, no evidence has investigated a possible relationship between mineral supplementation and vaccination side effects, particularly the SARS-CoV-2 vaccine. Moreover, no data are available concerning the significant association between side effects and mineral supplementation at the start of the vaccination cycle.

For the first time in our study, we observed that patients who take mineral supplementation (2.8%) are more likely to have no side effects than those who did not take it (97.2%) at the start of the vaccination cycle.

At the end of the vaccination cycle, mineral supplementation intake decreased by 1.1%.

Supplementation with *ω*-3 has been shown to influence the course of COVID-19. In general, Doaei et al. [48] observed that 1000 mg per day for 14 days of *ω*-3 administered to COVID-19 patients with severe symptoms led to an improvement in renal function. Additionally, Sedighiyan et al. [49] noted an improvement in some symptoms of COVID-19 (including pain and fatigue) and a reduction in PCR and ESR levels after supplementation with 2000 mg daily for two weeks.

However, our study is the first that investigate a possible relationship between *ω*-3 supplementation and vaccination side effects. In fact, regarding the significant association between side effects and *ω*-3 supplementation at the start of the vaccination cycle, we observed that patients with *ω*-3 supplementation (2.6%) are more likely to have no side effects than those who did not take it (97.4%) at the start of the vaccination cycle. At the end of the vaccination cycle, *ω*-3 supplementation decreases by 0.2%.

It has been observed that intestinal dysbiosis can influence the adverse effects of COVID-19 vaccines; therefore, by improving the intestinal microbiota, greater efficacy of the vaccine and a reduction of side effects are obtained [50]. It has also been found that the microbiota can play a crucial role in modulating the immune system and improving or reducing the response to vaccines according to their composition [29]. 

In the clinical study of Mak et al., the effect of a *Bifidobacteria*-based probiotic on reducing the adverse effects of the COVID-19 vaccine was analyzed in elderly patients with type 2 diabetes [51]. Indeed, in the management of COVID-19, *Bifidobacteria* seem to promote the efficacy of vaccines against SARS-CoV-2 [52]. Merra et al. [53] highlighted the importance of supplementation with probiotics of different compositions, depending on the nature of the dysbiosis, to positively influence the course of COVID-19.

In our study, at the start of the vaccination cycle, 3.5% of the participants were supplemented with probiotics. On the contrary, at the end of the vaccination cycle, only 2.3% of them took probiotic supplementation.

The main limitation of our study is its small sample that we may need to enlarge to have more heterogeneity, including other populations besides Italy.

Another limitation is the higher prevalence of women (71.3%) among survey participants. According to the National Institute of Statistics [54], this is possibly related to their greater participation in social media and because they spend more time on the web and social networks informing themselves about aspects concerning their health compared to men [55]. 

A strength of our study is the selection of the sample population without bias, due to the use of different dissemination channels. Furthermore, for the first time, we investigated the relationship between vaccine side effects and immunonutrients supplementation.

## 5. Conclusions

The results of the present study suggest that food supplements reduce the side effects of COVID-19 vaccinations in the general population during the vaccination cycle. At the start of the vaccination cycle, the most consumed supplements were found to be pre- and pro-biotics, with a reversal in favor of vitamin supplements (vitamins D, E, C, and B) at the end of the vaccination cycle. At the start of the vaccination cycle, no significant association was observed between vaccination side effects and the presence of comorbidities. At the start and end of the vaccination cycle, a significant correlation was observed between vaccine side effects and specific supplement intakes, demonstrating how supplementation can effectively assist vaccines in increasing host immune defenses and reducing side effects.

## Figures and Tables

**Table 1:** Participants’ general characteristics and anthropometrics.

	Whole Sample (N = 776)
Females	553 (71.3)
Males	223 (28.7)
Weight (kg)	69 ± 15
Height (cm)	168 ± 9
BMI (kg/m^2^)	24 ± 5
Sedentary lifestyle	313 (40.3)
Active lifestyle	463 (59.7)
Age (years)	43 ± 18
Groups by age	
18–24 years	65 (8.4; M 12.1)
25–42 years	335 (43.2; M 45.6)
43–60 years	274 (35.3; M 30.9)
Over 60	102 (13.1; M 19.7)
Affected by COVID-19	273 (35.2)
Vaccinated for COVID-19	776 (100)
Groups of vaccinated	
One dose	11 (1.4)
Two doses	139 (17.9)
Three doses	626 (80.7)

Values are expressed as mean and standard deviation (M ± SD) for continuous variables or as number and percentage (n (%)) for categorical variables. Abbreviation: BMI, body mass index.

**Table 2:** Supplemental intakes.

	Pre- and Probiotics	Vitamins D, E, C, and B	Zinc, Selenium, and Copper	Omega-3
1st dose	775 (99.9%)	208 (26.8%)	36 (4.6%)	20 (2.6%)
3rd dose	20 (2.6%)	194 (25.0%)	27 (3.5%)	19 (2.4%)

The main intakes distribution at the start (1st dose) and end (3rd dose) of the vaccination cycle. Values are expressed as numbers and percentages (n (%)) for categorical variables.

**Table 3:** Correlation between COVID-19 symptoms and comorbidities.

		COVID-19 Symptoms	Comorbidities
COVID-19 symptoms	Correlation coefficient	1.000	−0.08 *
	Sig. (2-code)		0.03
	N	776	770
Comorbidities	Correlation coefficient	−0.08 *	1.000
	Sig. (2-code)	0.03	
	N	770	770

The Spearman correlation was performed. Statistical significance was attributed as * *p* < 0.05 (2-code).

**Table 4:** Correlation between vaccination side effects, supplement intake, and comorbidities at the start of the vaccination cycle.

		Comorbidities	Vaccination Side Effects	Supplement Intake
Comorbidities	Correlation coefficient	1	0.03	0.07 *
	Sig. (2-code)		0.38	0.047
	N	770	770	770
Vaccination side effects	Correlation coefficient	−0.03	1.000	−0.001
	Sig. (2-code)	0.38		0.98
	N	770	776	776
Supplement intake	Correlation coefficient	0.07 *	−0.001	1.000
	Sig. (2-code)	0.047	0.98	
	N	770	776	776

The Spearman correlation was performed. Statistical significance was attributed as * *p* < 0.05 (2-code).

**Table 5:** Correlation between vaccination side effects, supplement intake, and comorbidities at the end of the vaccination cycle.

		Comorbidities	Vaccination Side Effects	Supplement Intake
Comorbidities	Correlation coefficient	1.000	0.008	0.06
	Sig. (2-code)		0.82	0.12
	N	770	770	770
Vaccination side effects	Correlation coefficient	0.008	1.000	0.59
	Sig. (2-code)	0.82		0.000 **
	N	770	776	776
Supplement intake	Correlation coefficient	0.06	0.59	1.000
	Sig. (2-code)	0.12	0.000 **	
	N	770	776	776

The Spearman correlation was performed. Statistical significance was attributed as ** *p* < 0.005 (2-code).

**Table 6:** Association between supplement intake and individual vaccination side effects, at the start, during, and at the end of the vaccination cycle.

	Supplement Intake		
	At the Start of the Vaccination Cycle	During the Vaccination Cycle	At the End of the Vaccination Cycle
Side Effects	*p*-Value	*p*-Value	*p*-Value
Pain in the injection site	0.71	0.80	0.48
Tiredness	0.53	0.58	0.76
Headache	0.30	0.78	0.40
Articular and muscle pains	0.14	0.30	0.24
Fever	0.73	0.25	0.10
Nausea	0.99	0.57	0.04 *
Diarrhea	0.007 *	0.10	0.001 **
Enlarged lymph nodes	0.53	0.052	0.98
Allergic reactions	0.38	0.48	0.99

The Chi-square test was performed. Statistical significance was attributed as * *p* < 0.05, ** *p* < 0.005.

**Table 7:** Association between vaccination side effects and individual supplement intakes at the start and end of the vaccination cycle.

	Side Effects	
	At the Start of the Vaccination Cycle	At the End of the Vaccination Cycle
Supplementation	*p*-Value	*p*-Value
Omega-3	0.001 **	0.29
Vitamins (D, E, C and B)	0.80	0.005 *
Minerals (zinc, selenium and copper)	0.02 *	0.28
Biotics (prebiotics and probiotics)	0.17	0.41

The Chi-square test was performed. Statistical significance was attributed as * *p* < 0.05, ** *p* < 0.005.

## Data Availability

The data presented in this study are available on request from the corresponding author.

## Appendix A

In Table A1, the questionnaire is reported.

**COVID-19 vaccines and supplements**

At the start of April 2020, the Section of Clinical Nutrition and Nutrigenomics, Department of Biomedicine and Prevention of the University of Rome Tor Vergata, within the scientific project EHLC-COVID-19, decided to investigate the change in Italians’ eating habits and lifestyle during Phase 1 of the pandemic caused by the new coronavirus called SARS-CoV-2.

After almost 3 years, the same research group, “Sustainable Nutrition for you—SuN4” wants to continue the scientific investigation to understand, following the COVID-19 vaccination campaign and the current situation on the health status of COVID-19-infected citizens, a possible relationship between supplementation and vaccine side effects.

If you are over 18, do not use drugs, do not have psychotic disorders, are not undergoing treatment for neoplastic diseases, are not pregnant or breastfeeding, and do not have HIV/AIDS, the SuN4U research team asks that you take a few minutes to complete the following questionnaire.

Your answers are extremely important, and by filling out the questionnaire, you accept that your answers will be used for research purposes. By consenting to the administration of the questionnaire, you also agree to fill it out only once.

The completion of the questionnaire can be performed subject to consent to the processing of data covered and protected by the Italian privacy law and the GDPR—General Data Protection Regulation—enforced in all European Union countries since 25 May, 2018 (general data protection regulation, officially EU Regulation No. 2016/679).

**Table A1:** Questionnaire.

Questions	Answers
Personal Data	
Sex at birth	Female/Male.
Age	Age in Numbers.
Region of residence	Valle d’Aosta (North-West Italy) Piedmont (North-West Italy) Lombardy (North-West Italy) Ligurian (North-West Italy) Trentino-South Tyrol (North-East Italy) Friuli-Venezia Giulia (North-East Italy) Veneto (North-East Italy) Emilia-Romagna (North-East Italy) Tuscany (Central Italy) Marche (Central Italy) Umbria (Central Italy) Lazio (Central Italy) Abruzzo (Southern Italy) Molise (Southern Italy) Campania (Southern Italy) Apulian (Southern Italy) Basilicata (Southern Italy) Calabria (Southern Italy) Sicily (Insular Italy) Sardinia (Insular Italy)
County of residence	Aosta (AO) Alexandria (AL) Asti (AT) Biella (BI) Cuneo (CN) Novara (NO) Turin (TO) Verbano-Cusio-Ossola (VB) Vercelli (VC) Bergamo (BG) Brescia (BS) Como (CO) Cremona (CR) Lecco (LC) Lodi (LO) Mantova (MN) Milan (MI) Monza e della Brianza (MB) Pavia (PV) Sondrio (SO) Varese (VA) Genoa (GE) Imperia (IM) La Spezia (SP) Savona (SV) Bolzano (BZ) Trento (TN) Gorizia (GO) Pordenone (PN) Trieste (TS) Udine (UD) Belluno (BL) Padova (PD) Rovigo (RO) Treviso (TV) Venice (VE) Verona (VR) Vicenza (VI) Bologna (BO) Ferrara (FE) Forlì-Cesena (FC) Modena (MO) Parma (PR) Piacenza (PC) Ravenna (RA) Reggio Emilia (RE) Rimini (RN) Arezzo (AR) Florence (FI) Grosseto (GR) Livorno (LI) Lucca (LU) Massa-Carrara (MS) Pisa (PI) Pistoia (PT) Prato (PO) Siena (SI) Ancona (AN) Ascoli Piceno (AP) Fermo (FM) Macerata (MC) Pesaro e Urbino (PU) Perugia (PG) Terni (TR) Frosinone (FR) Latina (LT) Rieti (RI) Rome (RM) Viterbo (VT) Chieti (CH) L’Aquila (AQ) Pescara (PE) Teramo (TE) Campobasso (CB) Isernia (IS) Avellino (AV) Benevento (BN) Caserta (CE) Napoles (NA) Salerno (SA) Bari (BA) Barletta-Andria-Trani (BT) Brindisi (BR) Foggia (FG) Lecce (LE) Taranto (TA) Matera (MT) Potenza (PZ) Catanzaro (CZ) Cosenza (CS) Crotone (KR) Reggio Calabria (RC) Vibo Valenzia (VV) Agrigento (AG) Caltanissetta (CL) Catania (CT) Enna (EN) Messina (ME) Palermo (PA) Ragusa (RG) Siracusa (SR) Trapani (TP) Cagliari (CA) Nuoro (NU) Oristano (OR) Sassari (SS) Sud Sardegna (SU).
Current employment	Study Job Unemployed Retiree
Degree	Primary School Lower Secondary School Upper Secondary School Graduation Master’s or Doctorate
How is your lifestyle?	Sedentary Not very active Moderately active Very active
Do you practice any sport(s)?	No Yes, aerobic sports Yes, anaerobic sports Yes, both aerobic and anaerobic sports
Do you currently smoke?	No Yes, <5 cigarettes/day Yes, >5 cigarettes/ day
Do you have any pathologies?	No Yes, I have respiratory pathology (pulmonary emphysema, chronic obstructive pulmonary disease (COPD), asthma, bronchiectasis, pulmonary fibrosis, OSAS (obstructive sleep apnea syndrome), and respiratory failure). Yes, I have cardiovascular diseases (angina pectoris, myocardial infarction, heart failure, ictus, and peripheral vascular disease). Yes, I have metabolic pathologies (dyslipidemia, diabetes, hyperglycemia, insulin resistance, and gout). Yes, I have neurological pathology (Alzheimer‘s disease, Parkinson’s disease, migraine, epilepsy, headaches, multiple sclerosis (SM), amyotrophic lateral sclerosis (SLA), and other autoimmune diseases). Yes, I have renal pathology. Yes, I have a tumor pathology. Yes, I have three or more pathologies among those listed. Yes, I have fewer pathologies than those listed.
**Anthropometrics information**	
Weight	Weight in kilograms (Kg)
Height	Height in centimeters (cm)
**COVID-19 infection and immune response**	
Did you contract COVID-19?	NoYes, I contracted COVID-19 before having the vaccination. Yes, I contracted COVID-19 after having the first vaccination. Yes, I contracted COVID-19 after having the second vaccination. Yes, I contracted COVID-19 after having the third vaccination.
Did you have any symptoms during COVID-19?	Asymptomatic Mild symptoms (cold, diarrhea) Flu symptoms (fever, cough, respiratory distress) Pneumonia.
Do you have any differences from your previous state of health after recovering from COVID-19?	Yes/No
**COVID-19 vaccination and supplementation**	
Did you get vaccinated for COVID-19?	Yes/No
How many doses?	1 2 3
Which vaccine did you receive at the first dose?	Pfizer Moderna AstraZeneca Jonson & Jonson
Did you have any side effects on the first dose?	No Pain at the injection site Tiredness Headache Muscle aches Chills Articular pains Diarrhea Fever Nausea Enlarged lymph nodes Allergic reactions with skin rashes or itching Other
How many days did the side effects last?	0 1 2 3 >4
Did you take the supplements?	No Probiotics Prebiotics (inulin, FOS) Vitamin D Vitamin C Vitamin E B-complex vitamins Zinc Selenium Copper Omega-3 L-arginine L-glutamine Folic acid Lactoferrin
For how many days?	I did not hire them. Yes, I only took them a week before the vaccination. Yes, I only got them the week after the vaccination. Yes, I took them both the week before and the week after the vaccination.
Who prescribed it for you?	No, I did not hire them. Self-prescription Nutritionist Doctor of general medicine Other.
Did you obtain the second dose of the vaccine?	Yes/No
Which vaccine did you receive at the second dose?	Pfizer Moderna AstraZeneca Jonson & Jonson.
Did you have any side effects on the second dose?	No Pain at the injection site Tiredness Headache Muscle aches Chills Articular pains Diarrhea Fever Nausea Enlarged lymph nodes Allergic reactions with skin rashes or itching Other
How many days did the side effects last?	0 1 2 3 >4
Did you take the supplements?	No Probiotics Prebiotics (inulin, FOS) Vitamin D Vitamin C Vitamin E B-complex vitamins Zinc Selenium Copper Omega-3 L-arginine L-glutamine Folic acid Lactoferrin
For how many days?	I did not hire them. Yes, I only took them in the week before the vaccination. Yes, I only got them a week after the vaccination. Yes, I took them both the week before and the week after the vaccination.
Who prescribed it for you?	No, I did not hire them. Self-prescription Nutritionist Doctor of general medicine Other
Did you obtain the third dose of the vaccine?	Yes/No
Which vaccine did you receive at the third dose?	Pfizer Moderna AstraZeneca Jonson & Jonson
Did you have any side effects on the third dose?	No Pain at the injection site Tiredness Headache Muscle aches Chills Articular pains Diarrhea Fever Nausea Enlarged lymph nodes Allergic reactions with skin rashes or itching Other
How many days did the side effects last?	0 1 2 3 >4
Did you take the supplements?	No Probiotics Prebiotics (inulin, FOS) Vitamin D Vitamin C Vitamin E B-complex vitamins Zinc Selenium Copper Omega-3 L-arginine L-glutamine Folic acid Lactoferrin
For how many days?	I did not hire them. Yes, I only took them in the week before the vaccination. Yes, I only got them a week after the vaccination. Yes, I took them both the week before and the week after the vaccination.
Who prescribed it for you?	No, I did not hire them. Self-prescription Nutritionist Doctor of general medicine Other.
Did you receive the antibody dosage at least 21 days after one of the vaccine doses?	No, I did not. Yes, I did, but the antibodies were absent. Yes, I did, and the antibodies were present.
